# Acute Abdomen due to Small Bowel Obstruction by Ascaris Lumbricoides in an 18‐Year‐Old Male: A Case Report and Review of the Literature

**DOI:** 10.1002/ccr3.70524

**Published:** 2025-05-20

**Authors:** Seyed Abbas Fatemiyoun, Farzad Rafiei, Seyed Shahab Adin Sheikhalishahi

**Affiliations:** ^1^ Department of General Surgery Shahid Sadoughi University of Medical Sciences Yazd Iran; ^2^ Student Research Committee Shahid Sadoughi University of Medical Sciences Yazd Iran

**Keywords:** acute abdomen, Ascaris lumbricoides (AL), case report, intestinal obstruction (IO), soil‐transmitted helminths (STH)

## Abstract

Intestinal obstruction caused by Ascaris lumbricoides (AL) is rare but clinically significant, particularly in endemic regions. We report an 18‐year‐old Afghan male who presented with a two‐month history of intermittent right lower quadrant pain, worsening over the past week with anorexia, constipation, nausea, and fatigue. Examination revealed generalized abdominal tenderness, involuntary guarding, and silent bowel sounds, suggesting an acute abdomen. Initial diagnosis was complicated appendicitis. After stabilization, laparotomy revealed a small bowel obstruction caused by a mass of AL, which was relieved via enterotomy and bowel repair. An incidental appendectomy was performed. Postoperatively, the patient was treated with albendazole and mebendazole. Bowel function returned by postoperative day three, and he was discharged on day five in stable condition. At one‐week follow‐up, he was asymptomatic with no complications. This case highlights the diagnostic challenges of parasitic infections, especially in non‐endemic areas, and emphasizes the importance of early imaging, timely surgical intervention, and comprehensive management. Clinicians should maintain a high index of suspicion for parasitic causes in bowel obstruction cases, particularly in endemic populations, to ensure prompt diagnosis and optimal outcomes.

**Abbreviations**
AL: Ascaris lumbricoides
BD: bis in die (twice a day)
CBC: complete blood count
Cr: creatinine
CT scan: computed tomography
D/C: discharge
Diff: differential white blood cell count
ED: emergency department
IO: intestinal obstruction
IV: intravenous
LFT: liver function test
LLQ: left lower quadrant
MCH: mean corpuscular hemoglobin
MCHC: mean corpuscular hemoglobin concentration
MCV: mean corpuscular volume
MHC: histocompatibility complex
MRI: magnetic resonance imaging
Na: sodium
POD: postoperative day
RBC: red blood cell
RLQ: right lower quadrant
SBO: small bowel obstruction
SGOT: serum glutamic‐oxaloacetic transaminase (AST)
SGPT: serum glutamic‐pyruvic transaminase (ALT)
STH: soil‐transmitted helminths
TB: tuberculosis
TDS: ter die sumendus (three times a day)
USG: ultrasonography
WBC: white blood cell
WHO: World Health Organization

**Summary**

- Parasitic infections like AL should be considered in bowel obstruction, especially in endemic, travel‐associated, and low socio‐economic cases, to avoid misdiagnosis and delays in treatment.
- Recognizing subtle but severe signs of obstruction, such as silent bowel sounds and involuntary guarding, is crucial for timely surgical intervention and preventing complications.

## 1 Introduction

Ascariasis, caused by the helminth AL, is a prevalent soil‐transmitted helminthic infection, particularly in regions with inadequate sanitation and hygiene practices [1]. While many infections are asymptomatic, heavy infestations can lead to serious complications, including intestinal obstruction, biliary colic, pancreatitis, and malnutrition [2]. Intestinal obstruction is a particularly severe manifestation, often necessitating prompt medical or surgical intervention [3]. Although ascariasis is commonly associated with pediatric populations in endemic areas, the occurrence of significant complications such as intestinal obstruction in young adults, especially in non‐endemic regions, is relatively rare [2]. This case report details an 18‐year‐old male presenting with acute abdomen due to small bowel obstruction by AL infestation. The uniqueness of this case lies in the patient's presentation with acute abdomen due to Ascaris, their age, the prolonged pain duration, and the successful surgical management employed. Reporting this case is crucial as it underscores the need for clinicians to consider parasitic etiologies in the differential diagnosis of acute abdomen, even in demographics and regions where such infections are uncommon. Furthermore, it contributes to the limited literature on adult presentations of ascariasis‐induced intestinal obstruction, enhancing understanding and awareness of this condition.

## 2 Case History/Examination

An 18‐year‐old Afghan male from a low socioeconomic background with low educational levels presented to the emergency department of Ali Ebn Abitaleb Hospital in Rafsanjan, Iran, on January 6, 2023, with complaints of abdominal pain. The patient's abdominal pain started approximately 2 months ago, initially intermittent and predominantly in the RLQ. He had visited outpatient clinics multiple times and received medical treatment. However, in the past week, his pain became persistent and worsened, forcing him to seek emergency care. Over the same period, he also had anorexia and had no bowel movements. He experienced nausea but denied vomiting. The patient had no known underlying medical conditions and no history of previous abdominal surgery. His drug history was unremarkable except for the occasional use of analgesics for pain relief. He reported no significant family history of medical conditions. The patient reports fatigue, anorexia, abdominal pain, nausea, and constipation. He denies fever, chills, vomiting, diarrhea, hematochezia, cough, dyspnea, or chest pain.

In physical examination the patient appeared pale, dehydrated, and malnourished. He was thin with a lean body mass, had no signs of respiratory distress, and had dry oral mucosa. The patient was stable with blood pressure: 120/85 mmHg; heart rate: 62 bpm; respiratory rate: 18 breaths per minute; temperature: 36.6°C; oxygen saturation: 99% on room air. In abdominal examination generalized tenderness was noted, along with involuntary guarding. There was no distension, and bowel sounds were silent. The rectum was empty and there were not any palpable masses. Respiratory examination revealed bilateral air entry with no added sounds. Cardiovascular examination revealed normal S1 and S2 with no murmur, and central nervous system examination revealed no neurological deficits. The initial clinical diagnosis was complicated appendicitis.

## 3 Investigations and Treatment

**FIGURE 1:**
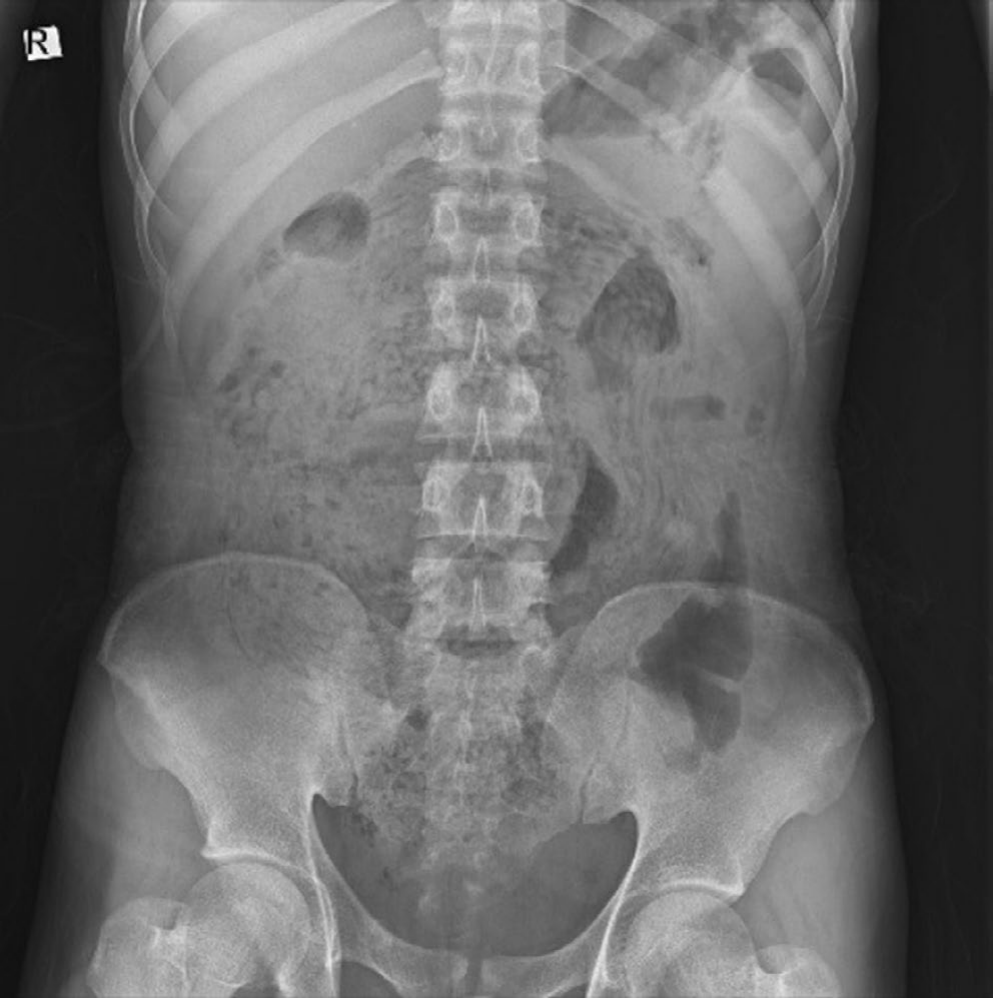
Abdominal X‐ray: The abdominal X‐ray demonstrates multiple linear, serpiginous, and tubular opacities, resembling the classic “railway track sign”, which is characteristic of intestinal ascariasis. This appearance is caused by the presence of multiple Ascaris worms aligned within the bowel loops. Additionally, the image reveals dilated bowel loops, suggesting SBO. There is no clear evidence of free air under the diaphragm, which would indicate perforation. No significant air‐fluid levels are apparent, but the bowel pattern is consistent with a mechanical obstruction due to worm burden.

**FIGURE 2:**
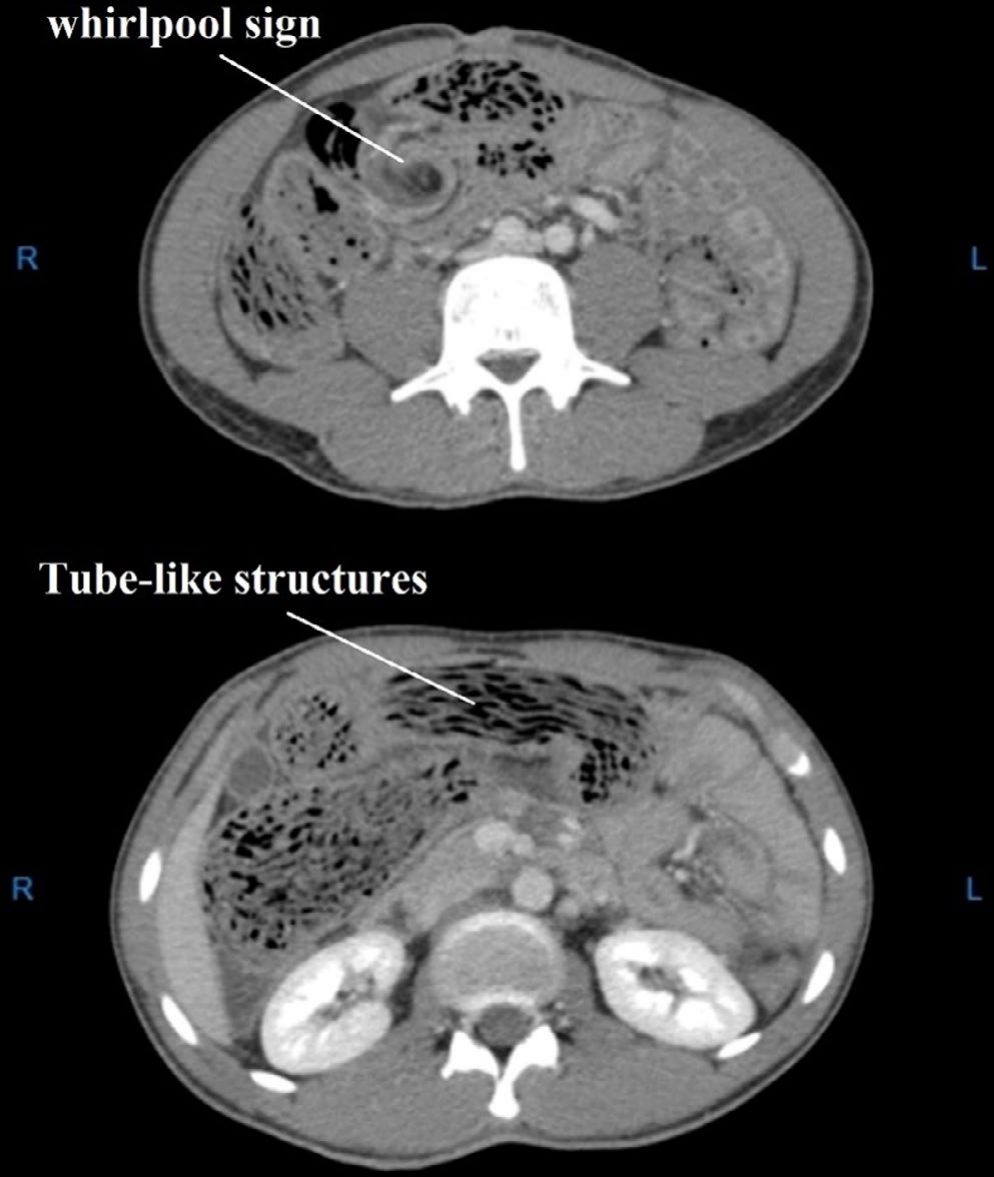
These axial CT images of the abdomen demonstrate characteristic findings of intestinal ascariasis with small bowel obstruction: “Tube‐like structures” within the intestinal lumen, which represent bundles of Ascaris worms. These appear as elongated, serpentine, non‐enhancing structures within the bowel loops. A “whirlpool sign” is observed in one section, indicative of a twisting of bowel loops, suggesting volvulus or impending obstruction due to the large worm burden. The findings are highly suggestive of intestinal ascariasis causing mechanical small bowel obstruction. The presence of a whirlpool sign raises concern for possible volvulus, which requires urgent intervention.

Laboratory tests including CBC, Diff, BUN, Cr, Na, K, BS, and LFT were ordered, with results summarized in Table 1. Additionally, the patient underwent a chest X‐ray and abdominal X‐rays (upright and supine views) in the emergency department (Figure 1). A surgical consultation was obtained, and an abdominopelvic computed tomography (CT) with IV contrast was requested (Figure 2). Preoperative diagnosis was bowel obstruction secondary to ascariasis.

**TABLE 1:** Laboratory tests.

CBC		WBC diff				LFT	
WBC	15,900/cumm	Neutrophil	83.0%	BS	151 mg/dL	Amylase	48 U/L
RBC	6.04 Mil/cumm	Lymphocyte	9.5%	Urea	30 mg/d	SGOT	31 U/L
Hemoglobin	17.9 g/dL	Monocyte Eosinophil	5.7%	Creatinine Sodium	0.84 mg/dL	SGPT	40 U/L
Hematocrit MCV	50.3%	Basophil	1.6%	Potassium	144 meq/L	Alkalin ph	326 U/L
MCH	83.3 FL	Lymphocyte	0.2%	Urea	4.0 meq/L		
MCHC	30.1 Pg						
Platelets	36.2 g/dL						
RBC	273,000/cumm						

The patient presented with symptoms of abdominal pain, nausea, anorexia, and constipation. On physical examination, findings included generalized abdominal tenderness, involuntary guarding, and silent bowel sounds, which are highly suggestive of intestinal obstruction. Potential underlying causes of the obstruction include tumors, volvulus, and congenital adhesions. Although the pain was initially intermittent and localized to the RLQ, raising the possibility of appendicitis, the progression to generalized symptoms makes this less likely. Additionally, perforated peptic ulcer, hernia (incarcerated or strangulated), and tuberculosis (abdominal TB) were included in the differential diagnosis.

**FIGURE 3:**
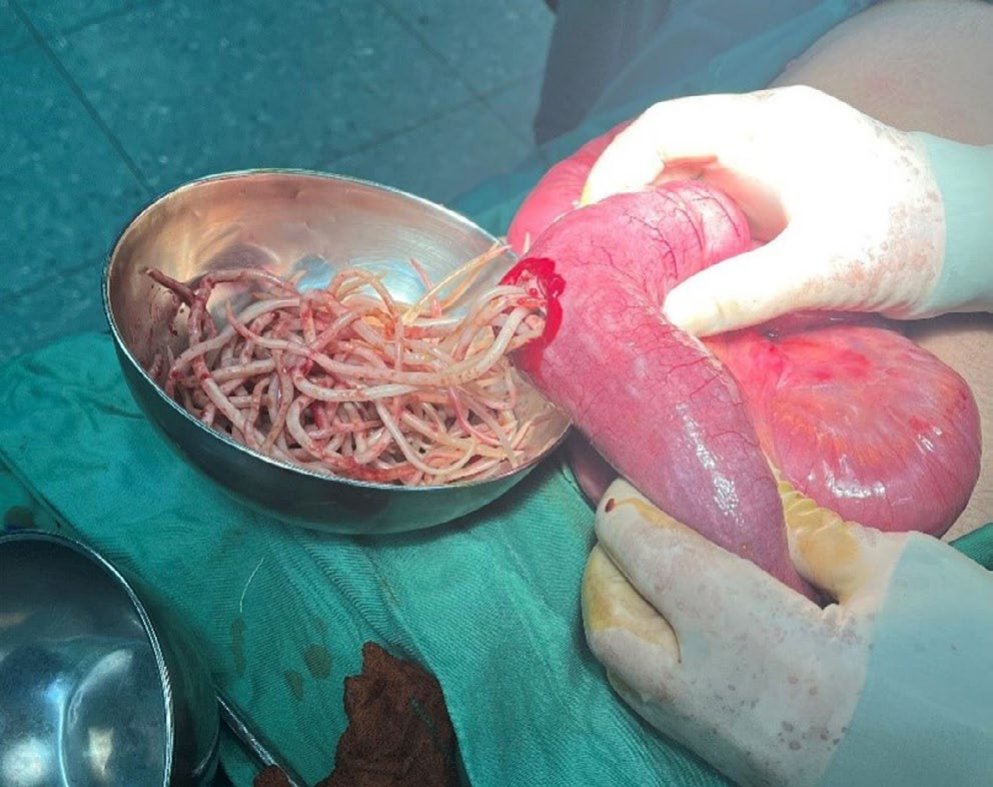
Enterotomy and removal of parasite manually.

**FIGURE 4:**
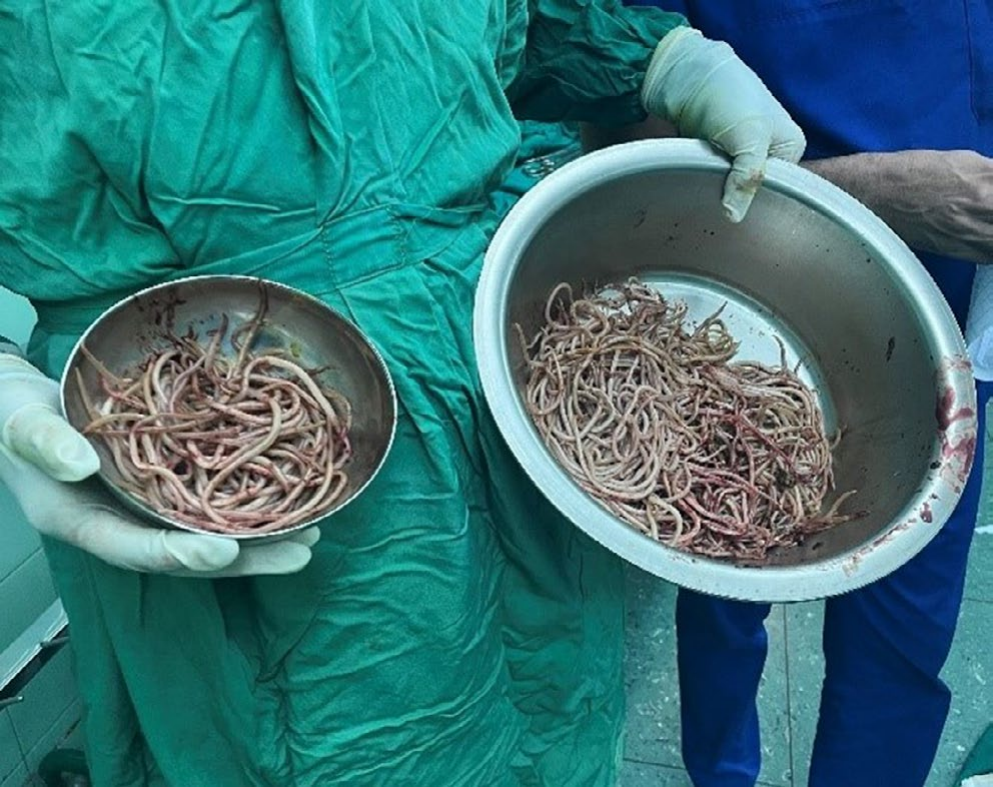
Multiple Ascaris worms expelled from the patient's intestine, highlighting a severe parasitic infection.

The patient underwent preoperative management, including fluid and electrolyte resuscitation to ensure stability. Prophylactic antibiotics, specifically metronidazole (500 mg) and ceftriaxone (1 g), were administered to prevent infection. Symptomatic management was provided with ondansetron for nausea, apotel for pain relief, and ketorolac. After these preparations, the patient was transferred to the operating room for the planned surgical procedure. In the operating room under general anesthesia, the patient was positioned supine, prepped, and draped. A lower midline laparotomy was performed; The small intestine from the terminal ileum up to 100 cm proximally was found to be distended and firm. A longitudinal enterotomy was made 40 cm from the cecum, revealing a large number of Ascaris worms obstructing the bowel lumen (Figures 3 and 4). The worms were extracted, and the obstruction was relieved. The remaining abdomen was explored; the colon was collapsed; more worms were observed proximal to the obstruction, extending up to 1 m from the terminal ileum, but they had not caused a complete obstruction; the small intestine was repaired using silk sutures. An incidental appendectomy was performed due to the appendix being exposed. The abdomen was thoroughly irrigated, and the fascia and skin were closed with nylon sutures.

Postoperatively, the patient continued to experience abdominal pain. An infectious disease consultation was obtained, and a single stat dose of albendazole 200 mg was administered; continuation of antibiotic therapy was planned, along with vitamin A and C supplementation. A few hours after albendazole administration, the patient reported pain relief. In POD 2, mebendazole 100 mg BD for 3 days was prescribed. In POD 3, the patient was allowed oral fluids. He had his first bowel movement, passing several worms, and his abdominal pain improved significantly. In POD 4, a regular diet was initiated. Finally, in POD 5, the patient was discharged with cefixime 400 mg BD, metronidazole 250 mg TDS, and naproxen 250 mg BD. He was advised to follow up in 1 week at the outpatient clinic. The patient's leukocytosis gradually decreased, as shown in Table 2

**TABLE 2:** Leukocytosis trend from admission to discharge.

Leukocytosis trend	
Admission day/operation day	15,900/cumm
POD 1	13,560/cumm
POD 2	11,810/cumm
POD 3	11,670/cumm
POD 4	10,980/cumm
POD 5/discharge	10,500/cumm

## 4 Outcome and Follow‐Up

The patient returned to the outpatient clinic 1 week postoperatively, as instructed. He reported no abdominal pain and no further passage of worms. On examination, he appeared well‐hydrated, with no signs of infection or complications. His surgical site was healing well, and bowel function was normal. No post‐operative complications or adverse events were noted. Precautionary measures included continued anti‐helminthic therapy, nutritional support, and hygiene education to prevent reinfection. He was advised to follow a balanced diet, maintain proper sanitation, wash hands thoroughly before meals and after using the toilet, and avoid consuming raw or unwashed vegetables. To minimize the risk of reinfection, his family was also counseled on hygiene measures, including proper handwashing, safe food handling, and sanitation improvements such as ensuring access to clean water and proper disposal of human waste. Routine deworming for household members was recommended, along with follow‐up evaluations if symptoms developed.

## 5 Discussion

Ascariasis, caused by the parasitic roundworm AL, is one of the neglected tropical diseases of humans. Globally, the estimated prevalence of AL infection is 11%. approximately 732 million individuals were infected with Ascaris worldwide in 2021; however, analyses revealed a reduction in prevalence between the periods 2016 and 2021 compared to 2010 and 2015 [4]. The lower prevalence observed in countries such as Iran over the last decades is correlated with positive shifts in socio‐economic indicators [5]. Ascariasis prevalence is higher in children, and individuals residing in poor socio‐economic conditions, and low human development indices promote the spread of parasites. Most individuals harbor a low to moderate parasitic burden. Although often asymptomatic, heavy infestations can lead to severe complications, including intestinal obstruction, which is a significant cause of morbidity and mortality, especially in children [6]. Ascariasis‐associated intestinal obstruction is a critical clinical condition that requires diagnosis and intervention to prevent life‐threatening complications such as bowel perforation, volvulus, and peritonitis [1]. Despite ascariasis‐associated intestinal obstruction prevalence among school‐age children [7], this complication in an 18‐year‐old male, as presented in this case, is notable.

Human infection with AL occurs by ingesting embryonated eggs through contaminated food, water, or soil. In the small intestine, the eggs hatch into larvae. Ascaris larvae invade the intestinal mucosa and enter the bloodstream. They are transported by portal and systemic circulation to the liver and lungs where they mature further within the alveoli. After 2 weeks, larvae ascend the bronchial tree to the pharynx and are swallowed to re‐enter the gastrointestinal tract. they develop into ovigerous adult worms approximately 9–11 weeks post‐ova ingestion [6, 8]. Environmental conditions, individual behaviors, and host factors such as genetics and immunity polymorphisms collectively affect disease outcome [9]. Soil contamination with egg or larvae showed a negative correlation with water storage and positive correlations with dog presence and home‐to‐latrine distance [10]. Researchers have evaluated the humoral immune response, cytokine responses, and the involvement of the major histocompatibility complex (MHC) interactions in the Ascaris infection patterns [11].

Mostly AL infection is asymptomatic, but early migration of Ascaris larvae can elicit a range of inflammatory responses, with liver and lung involvement potentially leading to eosinophilic granulomas and infiltration. AL verminous pneumonia is characterized by wheezing, dyspnea, a non‐productive cough, and fever, with hemoptysis commonly seen in severe cases. Children are more vulnerable to these pulmonary complications [8]. Intestinal parasitism clinical features related to obstruction often present with nonspecific gastrointestinal symptoms such as crampy abdominal pain, vomiting, which may be bilious, abdominal distension, and constipation or absence of flatus. In some cases, worms may be visible in vomitus or passed through the rectum. Other complications, such as abscesses, ascending cholangitis, acute cholecystitis, acute pancreatitis, and rarely bowel perforation, can also occur in patients [12]. AL aggregation and obstruction mostly occur in the ileum; however, when adult worms enter the appendix, they can cause pain and gangrene at the appendix tip, with a clinical presentation similar to acute appendicitis [8]. In the present case, the patient was initially diagnosed with suspected complicated acute appendicitis due to the clinical presentation and physical findings. An incidental appendectomy was performed during the surgical exploration because the appendix was exposed. A similar case has been reported in Ethiopia, where an obstruction caused by AL was associated with acute appendicitis [13]. This data highlights the importance of considering ascariasis in the differential diagnosis of acute abdomen, particularly acute appendicitis in endemic areas. Similar cases of intestinal obstruction caused by AL are briefly summarized in Table 3.

**TABLE 3:** Summary of similar cases of intestinal obstruction caused by AL: age, gender, location, clinical features, diagnostic findings, and management outcomes. Males were more frequently represented. The majority of cases occurred in endemic regions such as Ethiopia, Cameroon, Somalia, India, and Indonesia, highlighting the geographic prevalence of AL. Common symptoms across cases included colicky abdominal pain, vomiting, and obstipation, consistent with intestinal obstruction. Ultrasonography (USG) findings such as parallel paired lines (railway track sign) and curvilinear hypoechoic lesions were frequently observed. The ileum was the most common site of obstruction where the lumen is narrower.

Case/reference	Age	Gender	Location	Symptoms	Physical examination	Imaging	Level of obstruction	Treatment	Outcome
Mbanga et al. [14]	4	Male	Cameroon (endemic)	Generalized abdominal pains, vomiting, obstipation	Abdominal distention, mild tenderness, dehydration	X‐ray: discrete air‐fluid levels	Ileum	Enterotomy and parasite removal	D/C
Messay et al. [15]	19	Male	Ethiopia (endemic)	Colicky abdominal pain, vomiting, Inability to pass stool and Gas	Ill, abdominal distention with active bowel sounds	USG: distended small bowel loops, Multiple linear curly hypoechoic lesions	Ileum (partial)	Conservative, anthelmintic	D/C
Elmi et al. [13]	6	Male	Somalia (endemic)	Abdominal pain, constipation, vomiting	Tenderness and rigidity in the mid‐abdomen, distention, dehydration	USG: Multiple pairs of curvilinear echogenic lines, X‐ray: Multiple loops of dilated bowel, CT; multiple massively dilated loops of small bowel. Multiple elongated, tubelike structures	Ileum	Enterotomy and parasite removal	D/C
Hailu et al. [16]	6	Female	Ethiopia (endemic)	Colicky periumbilical pain, vomiting	Abdominal distension and tenderness	USG: long segment ileocolic intussusception with a significant load of ascariasis, X‐ray: whirlpool in the right iliac fossa	Distal ileum	Enterotomy and parasite removal	D/C
Saini et al. [17]	4	Male	India (endemic)	Abdominal pain, vomiting, pica	Distention, Generalized tenderness, and rigidity in the center of the abdomen	X‐ray: multiple air‐fluid levels, USG: parallel paired lines	Small intestine	Enterotomy and parasite removal	D/C
Turyasiima et al. [18]	4	Female	Uganda (endemic)	Colicky periumbilical pain, vomiting	A palpable mass in the left paraumbilical area	USG: a round mass in the right iliac region	Ileum	Enterotomy and parasite removal	D/C
Abdellatif et al. [19]	12	Male	Egypt (endemic)	Colicky periumbilical pain; vomiting and constipation	Abdominal tenderness and rigidity in the central and mid‐abdomen	USG: parallel paired lines like “railway tracks”	Jejunum	Enterotomy and removal of parasite	D/C on the POD4
Perret and Simon [20]	8	Female	USA (non‐endemic)	Pain in RLQ, vomiting	Abdominal distention and a palpable mass in LLQ	None	Small intestine	Purgative enema	D/C
Birhanu et al. [21]	25	Female	Ethiopia (endemic)	Crampy periumbilical pain, vomiting, obstipation	Periumbilical distention; 6 by 5 cm soft mobile nontender mass on RLQ	X‐ray: dilated multiple air‐fluid levels	Ileum	Enterotomy and removal of parasite manually	D/C
Sulmiati et al. [22]	5	Male	Indonesia (endemic)	Abdominal pain; Vomiting, Obstipation	Tenderness and palpable mass in right hypochondriac	X‐ray: a view of mass whirlpool image, with air‐fluid level	Terminal ileum	Enterotomy and removal of parasite	D/C
Romano et al. [23]	75	Male	Italy (non‐endemic)	Abdominal pain; vomiting, obstipation	Abdominal distension and Tenderness	CT‐scan: multiple air‐fluid levels in the epigastrium and left hypochondriac	Ileum	Enterotomy and removal of parasite	D/C

Diagnosis is typically based on clinical suspicion, especially in endemic regions supported by imaging and laboratory findings. The gold standard for diagnosing ascariasis definitively relies on the visualization of Ascaris larvae in respiratory secretions or gastric aspirates. Identifying AL eggs in stool samples via microscopic examination is a reliable method for diagnosis. The X‐ray may show dilated bowel loops with air‐fluid levels suggesting the obstruction, while ultrasound can identify characteristic linear, echogenic structures with central hypoechoic tubes representing the worms. In cases where imaging is inconclusive, CT can provide detailed information about the obstruction with high sensitivity and specificity. It may reveal characteristic findings such as “whirlpool” or “target” signs caused by worm masses [24]. Magnetic resonance imaging (MRI) can be a useful modality in cases with suspected hepatobiliary involvement [25].

The management of ascariasis‐associated obstruction typically involves a combination of medical and surgical approaches. Conservative management for partial obstruction includes anthelminthic therapy (albendazole or mebendazole) combined with nasogastric decompression and supportive care, including intravenous fluids and electrolytes. Surgical intervention in cases of complete obstruction, perforation, or failure of conservative management requires surgical removal of the worm bolus in addition to antibiotic therapy if a systemic infection is suspected. Techniques include enterotomy and manual extraction of worms. Follow‐up with anthelminthic therapy and education on preventive measures, such as improved hygiene and access to clean water, is essential to prevent recurrence. To monitor treatment effectiveness for ascariasis, after surgery or when the worm burden is high, collecting stool samples 2 weeks after treatment up to three times is recommended [1]. The findings from this case underscore the importance of early diagnosis and management of ascariasis to minimize mortality and avoid complications such as obstruction. Implementation of public health initiatives to reduce ascariasis through improved sanitation, access to clean water, and mass deworming programsis necessary.

## Author Contributions

**Seyed Abbas Fatemiyoun:** conceptualization, data curation, investigation, project administration, supervision, validation, visualization, writing – review and editing. **Farzad Rafiei:** writing – original draft, writing – review and editing. **Seyed Shahab Adin Sheikhalishahi:** writing – original draft, writing – review and editing.

## Ethics Statement

Consent to participate Written informed consent was obtained from the patient for publication of this case report and accompanying images. Consent for publication The patient has consented to the submission of the case report to the journal.

## Conflicts of Interest

The authors declare no conflicts of interest.

## Data Availability

The clinical data related to this case report are available on request from the corresponding author. The data are not publicly available due to privacy and ethical restrictions.
